# Editorial: Long non-coding RNAs in viral infections and immunity

**DOI:** 10.3389/fimmu.2023.1198979

**Published:** 2023-04-26

**Authors:** Saikat Boliar, Laura Prats-Mari, Puri Fortes

**Affiliations:** ^1^ Department of Microbiology and Immunology, College of Veterinary Medicine, Cornell University, Ithaca, NY, United States; ^2^ DNA and RNA Medicine Division, RNA Biology and Therapy Program, Center for Applied Medical Research (CIMA), University of Navarra (UNAV), Pamplona, Spain; ^3^ Cancer Center Clinica Universidad de Navarra (CCUN), Madrid, Spain; ^4^ Navarra Institute for Health Research (IdiSNA), Pamplona, Spain; ^5^ Spanish Network for Advanced Therapies (TERAV ISCIII), Madrid, Spain; ^6^ Liver and Digestive Diseases Networking Biomedical Research Centre (CIBERehd), Madrid, Spain

**Keywords:** lncRNAs, non-coding RNA, virus, infection, immunity

Long non-coding RNAs (lncRNAs) have emerged in recent years as the largest class of RNAs that are transcribed from the human genome (1, 2). However, until recently, these RNAs were disregarded as “junk”, due to their inability to produce functional proteins. But it is now evident that these lncRNAs can assume crucial roles in almost every aspect of biology, including viral pathogenesis and immunity (3–6). A collection of six articles encompassing both original research and reviews published in Frontiers in Immunology have delved into recent advances in this field (**Figure 1**).

**Figure 1:**
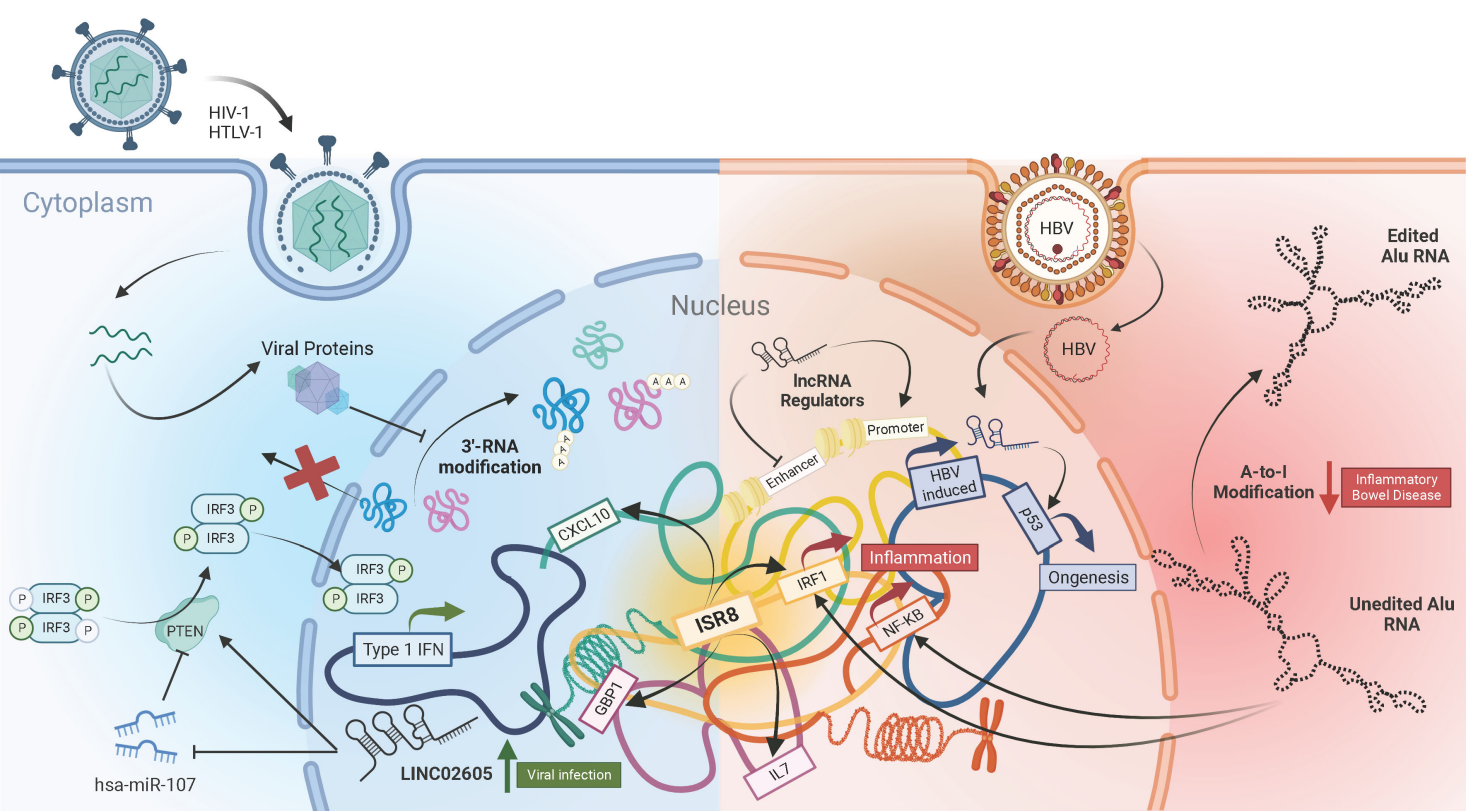
The complex landscape of lncRNAs in viral Infections and immunity.

Viruses, being obligatory intracellular pathogens, are dependent on host cell processes such as transcription and translation machinery for their replication. Viruses have also evolved to modulate those cellular pathways to subvert host anti-viral responses. One such mechanism, as detailed by Vijayakumar et al. in their review article, is by disrupting the 3’-processing and maturation of cellular mRNAs. This viral inhibition of pre-mRNA cleavage and polyadenylation results in generation of a new class of non-coding RNA transcripts called Downstream-of-Gene (DoG) that are retained in the nucleus and therefore aren’t translated into proteins. The authors describe how different classes of viruses such as Influenza, HSV-1, Enterovirus and HIV-1 affect the host mRNA maturation process and explore its implications on the host functions including anti-viral immune responses.

Infection with hepatitis B virus (HBV) which causes hepatocellular carcinoma, is a major public health concern globally. Samudh et al. provide an up-to-date review of the lncRNAs that have been implicated in HBV pathogenesis. They pointed out that the majority of lncRNAs associated with HBV infection promote oncogenesis, while only a few HBV infection-associated lncRNAs are suppressors of tumor growth. They describe the varied ways lncRNAs can modulate HBV replication, including removal of epigenetic silencing of the viral covalently closed circular DNA (cccDNA), translocation of transcription factors to the viral promoter and inhibition of anti-viral microRNAs. Many HBV-induced lncRNAs are also identified to interact with proteins such as p53, NPM1 and PGK1 to promote cellular proliferation and angiogenesis. The roles detailed in the review highlight the potential of modulating lncRNAs as an attractive curative avenue for HBV infection.

Transcription of non-coding RNAs isn’t limited to host cells but also extends to viruses. The review article by Toyoda and Matsuoka describes how retroviruses employ bi-directional transcription of their genomes to generate alternate, anti-sense viral RNA transcripts that aid in viral persistence and pathogenesis. Both HTLV-1 and HIV-1 produce an anti-sense transcript from promoters in the 3’-long terminal repeat (LTR) region of their genome. Inefficient polyadenylation of the anti-sense transcripts ensures their predominantly nuclear localization. The HTLV-1 encoded anti-sense transcript can promote immune escape and proliferation of the infected cells leading to their prolong survival and persistence. On the other hand, the anti-sense transcripts of HIV-1 negatively affect viral transcription from their 5’-LTR promoters, thereby facilitating latency. The article highlights the functional importance of virus-encoded non-coding RNAs and the necessity to study them in further detail.

Given such lncRNA-mediated modifications of the antiviral response, it is not surprising that cellular non-coding RNA sequences are strong regulators of the innate immune response in mammalian cells (7). A fascinating example are the highly abundant Alu sequences, which cover 10% of the human genome (8). Research from Aune et al. reveled that Alu forms dsRNAs that are sensed predominantly by RIG-I, leading to activation of the type I IFN synthesis and signaling pathways. RIG-I sensing requires the 5´-end PPP and the left arm of the Alu sequence. Healthy cells avoid Alu activation by several means, including A-to-I RNA editing by the adenosine deaminase ADAR. Only 5 to 8 edits in the 3´-end of the left arm of each Alu molecule, predicted to break a long dsRNA structure, are sufficient to block activation of the IFN response. Interestingly, small amounts of edited Alu block full IFN activation by non-edited Alu. Thus, the balance between edited and non-edited Alu is relevant for the response. This has therapeutic and clinical implications. In fact, loss of editing is associated with infections with influenza or coronavirus and the degree of loss is proportional to disease severity. Editing loss is also observed in immune diseases such as multiple sclerosis, ulcerative colitis or Chron´s disease. In these, the reduced number of common editing sites could be caused by an increase in negative regulators of A-to-I editing factors.

Specific lncRNAs also affect the antiviral response with simpler or more complex mechanisms. Xu et al. identified the LINC02605 that is induced by STAT and NF-κB signaling in infected cells and blocks viral replication. Mechanistically, LINC02605 increases the levels of PTEN, which promotes dephosphorylation of IRF3 at serine 97, nuclear IRF3 translocation and enhanced transcription of ISGs. Instead, IRF1-AS1/ISR8 is a good example of the high complexity that mammalian cells have developed to regulate the innate immune response (9). IRF1-AS1 gene, located antisense to the IRF1 coding gene, transcribes a lncRNA in response to type I IFN, IRFs or NF-κB activation (10). However, IRF1-AS1 *per se* does not regulate the innate response. Instead, IRF1-AS1 could mark the functionality of an enhancer located in the IRF1-AS1 locus. Thus, depletion or overexpression of IRF1-AS1 does not result in any immune-related phenotype, as reported by Barriocanal et al. It is the disruption of IRF1-AS1 locus what results in cells that are extremely susceptible to viral infection even in the presence of high doses of type I IFN: they fail to produce ISGs or inflammatory genes in response to IFNα or NF-κB activation. Surprisingly, this lack of response is only at genomic, but not at episomic level. IFNα and NF-κB promoters respond to these activators when they are in a plasmid but not when the same sequences are integrated into the genome. This highlights the enhancer nature of the regulation. Concordantly, transcription from these promoters is not restored with silencing inhibitors or by overexpression of inducers. Interestingly, transcriptomic analyses of IRF1-AS1-disrupted cells show that they are enriched in negative regulators such as EZH2, KAP1, XIST or several Zinc finger proteins. These findings have clinical relevance as IRF1-AS1 locus is populated with SNPs associated with chronic autoimmune and inflammatory diseases such as asthma, ulcerative colitis or Crohn´s disease. This points to the possibility that modifications in the IRF1-AS1 region alter enhancer functionality, resulting in profound deregulations of antiviral and inflammatory genes.

In summary, the work compiled in the special issue serves to highlight the importance of viral and cellular lncRNAs in the regulation of infections and immunity. Understanding the mechanisms that drive the functionality of these lncRNAs will be fundamental to develop novel therapies for the treatment of viral infections and immune diseases.

## Author contributions

SB and PF wrote the manuscript. LP-M generated the figure. All authors read and approved it for publication.
